# Perioperative Anesthetic Management of Kleine-Levin Syndrome: Current Evidence, Mechanistic Rationale, and a Proposed Practice Framework

**DOI:** 10.7759/cureus.114751

**Published:** 2026-08-18

**Authors:** Fareena Khan, Vikas Chauhan, Victoria Wang

**Affiliations:** 1 Anesthesiology, University of Mississippi Medical Center, Jackson, USA; 2 Medical School, University of Mississippi Medical Center, Jackson, USA

**Keywords:** anesthetic management, central hypersomnolence, delayed emergence, kleine-levin syndrome, narrative review, opioid-sparing analgesia, perioperative medicine, postoperative hypersomnolence, rare disease anesthesia, recurrent hypersomnia

## Abstract

Kleine-Levin syndrome is a rare disorder of central hypersomnolence characterized by relapsing-remitting severe sleepiness associated with cognitive impairment, derealization, apathy, behavioral change, and hyperphagia. Direct perioperative evidence remains limited and is insufficient to establish comparative safety, recurrence risk, or a preferred anesthetic technique. Reports with direct perioperative evidence described favorable immediate perioperative courses, but follow-up was heterogeneous, and the uniformly favorable published outcomes are susceptible to substantial publication bias. Most patients return to normal or near-normal function between episodes, although residual sleep, cognitive, behavioral, or autonomic symptoms persist in a subset. The perioperative period introduces factors that may obscure evaluation or disturb sleep-wake stability, including sleep disruption, physiologic stress, perioperative illness, and exposure to sedative and opioid medications. No specific perioperative trial data are available, so this narrative review synthesizes direct disease-specific evidence, mechanistic rationale, and established perioperative practice to propose a conservative, evidence-mapped framework. Disease-informed considerations include elective scheduling during a stable interepisodic period when feasible, documentation of baseline cognitive and behavioral status with collateral history, and treatment of suspected recurrence as a diagnosis of exclusion. Other considerations, including minimizing unnecessary sedatives and long-acting opioids, considering regional, neuraxial, or local techniques and opioid-sparing analgesia, using short-acting titratable agents, and applying anesthetic depth and neuromuscular monitoring, represent established perioperative practices adapted to this population. These considerations are conditional and hypothesis-generating, not formal guidelines.

## Introduction and background

Kleine-Levin syndrome (KLS) is a rare disorder, with an estimated prevalence of one to five cases per million, and it most commonly begins during adolescence with a relapsing-remitting course. Clinically, an episode may resemble a previously functioning adolescent or young adult suddenly sleeping for most of the day and, when awake, appearing cognitively slowed, apathetic, derealized, irritable, or behaviorally disinhibited [1-4]. Patients usually return to normal or near-normal function between episodes, although neuropsychological, functional imaging, and systematic symptom-assessment studies indicate that subtle abnormalities may persist in some patients during remission [1-9]. Male predominance is well recognized: a systematic review of 186 cases reported 68% male, while a later systematic study of 108 patients reported 78% male [2,10]. Because of its rarity, current knowledge is derived primarily from cohort studies, systematic reviews, and case reports rather than controlled trials.

The central perioperative challenge is that expected postoperative drug effects can resemble the disease itself. Residual anesthetics, opioid-related sedation or hypoventilation, postoperative delirium, sleep deprivation, hypercapnia, and pain may overlap with features of a KLS episode [11-20]. Because anesthetic agents influence hypothalamic, thalamocortical, and limbic arousal networks implicated in sleep-wake regulation, perioperative planning should emphasize both physiologic stability and a postoperative assessment that can distinguish pharmacologic effects from a KLS-like phenotype [11-17].

The available anesthetic literature consists of isolated reports rather than comparative studies. Five dedicated patient-level perioperative management reports have been published, comprising one combined spinal-epidural anesthetic and four general anesthesia cases [21-25]; one was published in letter format and was more abbreviated in presentation while still providing extractable patient-level perioperative data [25]. These reports demonstrate feasibility but cannot establish a preferred anesthetic technique, comparative safety, or the incidence of postoperative recurrence.

This narrative review aims to provide a transparent, citation-mapped framework for perioperative management of KLS by separating direct patient-level observation from mechanistic rationale and established perioperative principles. The proposed considerations are therefore conditional and hypothesis-generating, based on limited direct experience, current understanding of sleep-wake regulation, and general anesthetic practice applied to this rare population.

## Review

Literature search and narrative synthesis

This narrative review integrated the limited perioperative literature on KLS with selected contextual evidence from sleep medicine, anesthesiology, and perioperative care. PubMed/MEDLINE (MEDical Literature Analysis and Retrieval System Online) and Google Scholar were searched without a lower publication-date restriction through July 2026 using combinations of terms related to KLS, recurrent hypersomnia, anesthesia, perioperative care, surgery, sedation, delayed emergence, and postoperative somnolence. Reference lists and forward citations of relevant KLS reviews and perioperative reports were also examined. The principal PubMed search returned 10 records, identifying four of the five dedicated perioperative management reports; Google Scholar and citation tracking identified the remaining report.

Direct perioperative management evidence was defined as a publication in which anesthetic or perioperative management of a patient with established primary KLS was a substantive focus and sufficient patient-level information was available to characterize the technique, perioperative course, or postoperative outcome. Reports of secondary KLS, incidental anesthesia exposure without extractable management information, mixed hypersomnolence cohorts without separable KLS outcomes, conference abstracts lacking sufficient detail, and duplicates were excluded. Five dedicated reports met these criteria: one involving combined spinal-epidural anesthesia [21] and four involving general anesthesia [22-25]. For each eligible report, patient and disease characteristics, procedure, anesthetic technique and monitoring, perioperative course, and follow-up were extracted from the primary publication. Reporting completeness was appraised descriptively using the JBI Critical Appraisal Checklist for Case Reports [26], without a numerical quality score or exclusion threshold (domain-level results are provided in the Appendices).

Additional literature was selected purposively to contextualize KLS phenotype and treatment, sleep-wake and emergence neurobiology, perioperative medication management, anesthetic monitoring, and related hypersomnolence disorders. Guidelines, consensus statements, systematic reviews, and major cohort studies were prioritized when available. Evidence was synthesized descriptively. Because the direct evidence comprised only five heterogeneous, uncontrolled single-patient reports, statistical pooling was not feasible; no meta-analysis or formal certainty-of-evidence grading was performed. Contextual evidence was used to support biologic plausibility and established perioperative principles rather than to establish KLS-specific benefit.

Clinical phenotype and perioperative relevance

Symptoms during KLS episodes include excessive sleepiness, cognitive impairment, apathy, derealization, behavioral changes, mood disturbance, and variable hyperphagia or hypersexuality, consistent with current International Classification of Sleep Disorders, Third Edition, Text Revision (ICSD-3-TR) criteria [1-4,27]. Episodes typically begin abruptly and persist from two days to several weeks, followed by normal or near-normal function between episodes. Establishing the patient’s disease phase and baseline function before surgery is therefore essential to the interpretation of postoperative recovery.

A recent systematic assessment of consecutive patients using validated behavioral, hypersomnolence, and autonomic instruments, with input from relatives, documented frequent dysexecutive symptoms, hypersomnolence, and dysautonomia during episodes, together with residual symptoms in a subset of patients between episodes [9]. These findings support three elements of the framework proposed here: the value of collateral history, the documentation of a patient-specific cognitive and behavioral baseline rather than the assumption of complete interepisodic normality, and attention to autonomic and thermoregulatory features during perioperative care.

Recent work further underscores phenotypic and physiologic heterogeneity. Mixed episodes containing both hypersomnia and insomnia have been described as a marker of greater disease severity [28], while a 2026 actigraphy and melatonin study found largely preserved circadian markers during both remission and relapse, arguing against a uniform circadian-phase abnormality as the sole mechanism of KLS [29].

**Figure 1:**
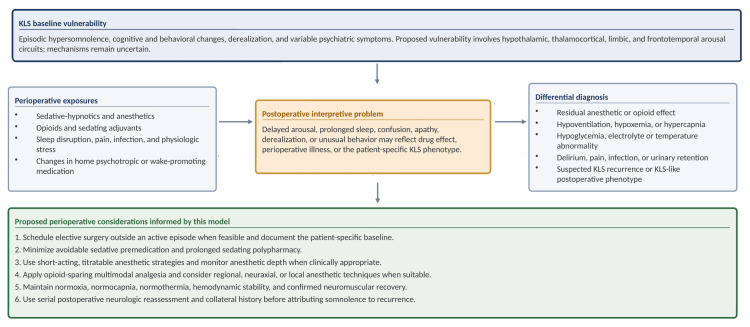
Proposed Perioperative Interaction Model in Kleine-Levin Syndrome This conceptual model illustrates how baseline KLS-related arousal network vulnerability and perioperative exposures may contribute to delayed arousal, prolonged sleep, confusion, derealization, apathy, or unusual behavior. Such findings should prompt systematic evaluation for residual anesthetic or opioid effects, hypoventilation, hypoxemia, hypercapnia, metabolic or temperature abnormalities, delirium, pain, infection, urinary retention, and other perioperative causes before recurrence is presumed. The model is mechanistic and hypothesis-generating and does not establish that anesthetic exposure causes a KLS episode. KLS: Kleine-Levin syndrome

Reported episode triggers include infection, sleep deprivation, alcohol exposure, head trauma, and psychological stress; however, their causal relationship remains uncertain, and perioperative recurrence has not been prospectively demonstrated [1-4]. Figure 1 depicts the proposed perioperative interaction model in KLS. The primary perioperative goal is not elimination of an unquantified recurrence risk but reduction of avoidable confounders and preservation of a clear postoperative assessment.

Direct perioperative evidence in KLS

The direct perioperative management record consists of five dedicated patient-level publications [21-25]. One was published in letter format [25] and is more abbreviated than the full case reports. All five provide sufficient perioperative management information for descriptive extraction and are summarized in Table 1. They establish feasibility in individual patients but cannot define the incidence of delayed emergence or recurrence, compare anesthetic techniques, or demonstrate that anesthesia does not precipitate recurrence. Older KLS literature contains isolated descriptions of anesthesia exposure or anesthesia-associated episodes, but without sufficient contemporary management detail for inclusion in the direct perioperative management set [2].

**Table 1:** Dedicated Patient-Level Perioperative Management Reports in Primary Kleine-Levin Syndrome BIS: bispectral index; BMI: body mass index; EEG: electroencephalography; GA: general anesthesia; KLS: Kleine-Levin syndrome; LMA: laryngeal mask airway; NMB: neuromuscular blockade; POD: postoperative day; TCI: target-controlled infusion; TIVA: total intravenous anesthesia; M: male; F: female

Study (Author, Year)	Patient age, sex/KLS Status	Procedure	Technique and Monitoring	Emergence/Course	Follow-Up	Influence and Limitation
Rehman et al., 2018 [21]	41 years, F/Remission (last episode three months prior)	Elective total abdominal hysterectomy	Combined spinal-epidural; no anxiolytic premedication; standard monitoring	Uneventful; motor block resolved by 3 hour; discharged POD4	Days 7, 14; no recurrence	Supports neuraxial feasibility outside an episode; does not address GA
Ben-Menachem and Winder, 2021 [22]	23 years, M/Three episodes in preceding three months	L5/S1 microdiscectomy	TIVA (entropy-guided propofol TCI); fentanyl; rocuronium/sugammadex	Brief disorientation, then oriented; met discharge criteria	4 weeks; no recurrence	First reported TIVA use in KLS; single case, no comparator
Fujita and Mizuta, 2022 [23]	22 years, F/Surgery during crisis interval	Third molar extraction	GA: propofol/remifentanil/rocuronium; EEG-depth + NMB monitoring; sugammadex	~10 minutes, EEG-confirmed	Not specified; no recurrence reported	Supports feasibility of monitored short-acting GA; single case
Rajaleelan et al., 2022 [24]	27years, F/No active daytime sleepiness	Endoscopic skull-base resection	GA; short-acting agents; depth monitoring; opioid-sparing multimodal analgesia	Favorable course	Sleep-medicine follow-up; no recurrence	Supports feasibility for a longer neurosurgical case; no comparator
Chowdhury et al.,2023 [25] (letter)	38 years, F/BMI 36, 22-year remission; psychiatric/thyroid/seizure comorbidity	Emergency corneal repair	Chronic medicine continued; non-pharm anxiolysis; titrated propofol/fentanyl/atracurium; LMA; BIS-titrated desflurane	Extubated fully awake; overnight observation	4 months; symptom-free	Illustrates non-pharm anxiolysis as premedication alternative; long remission limits generalizability

Rehman et al. reported combined spinal-epidural anesthesia for abdominal hysterectomy in a patient outside an acute episode [21]. The authors selected neuraxial anesthesia because of concern that anesthetic agents and narcotics might trigger or confound KLS symptoms and characterized general anesthesia as contraindicated; this reflected case-level opinion rather than established evidence; subsequent reports documented successful general anesthesia [22-25]. Ben-Menachem and Winder reported propofol-based total intravenous anesthesia with entropy monitoring for lumbar microdiscectomy in a patient with three episodes during the preceding three months, followed by an uneventful recovery and no hypersomnolence at four weeks [22]. Fujita and Mizuta described extraction of four third molars during a symptom-free interepisodic interval using propofol, remifentanil, rocuronium, desflurane, SedLine® monitoring (Masimo Corporation, Irvine, California, United States), neuromuscular monitoring, and sugammadex; emergence occurred within approximately 10 minutes, and the patient was discharged on postoperative day 4 [23].

Rajaleelan et al. described a seven-hour endoscopic skull-base procedure using sevoflurane-remifentanil anesthesia with entropy monitoring in a 27-year-old woman whose subjective sleepiness was minimal despite markedly reduced sleep latency on objective testing; she was fully awake at extubation and reported no worsening of KLS symptoms at four weeks [24]. Chowdhury et al. described emergency ophthalmic surgery under titrated desflurane anesthesia with bispectral index monitoring in a patient who had been asymptomatic for 22 years; parental presence and familiar music were used as non-pharmacologic anxiolytic measures, and she remained symptom-free at four months [25]. Across the reports, recurring management considerations included avoidance of unnecessary sedative premedication, titratable anesthetic exposure, and deliberate postoperative observation, but these practices were not tested comparatively.

These are favorable descriptions of feasibility rather than evidence of safety. All five reports described favorable immediate perioperative courses, but follow-up duration and ascertainment of later KLS symptoms were heterogeneous [21-25]. The evidence is highly susceptible to publication bias, selective outcome reporting, and the absence of any comparator group.

Descriptive JBI appraisal showed generally complete reporting of patient characteristics, anesthetic interventions, postoperative outcomes, and clinical lessons across the five reports. The principal reporting limitation was incomplete description of disease-specific diagnostic assessment in the earliest report. All five reports were retained because the appraisal was used to characterize reporting limitations rather than to generate a numerical quality score or determine eligibility [26].

The evidence informing the remainder of this review spans direct case-level observations, KLS disease literature, established perioperative guidance, and mechanistic or analog-disorder evidence. Table 2 summarizes the role and limits of inference for each source type.

**Table 2:** Evidence Sources and Limits of Inference in this Narrative Review This author-developed evidence map distinguishes direct observation from indirect inference. It is not a validated certainty-of-evidence grading system or guideline-development framework. KLS: Kleine-Levin syndrome

Evidence source	Role in the narrative synthesis	What it can reasonably support	Principal limitation
Direct KLS perioperative case reports (five patient-level publications: one neuraxial and four general anesthesia, one of which appeared in letter format)	Describe anesthetic technique, monitoring, emergence, and observed outcomes in named patients	Feasibility of neuraxial and general anesthesia in the individual patients reported	Cannot establish incidence, comparative safety, or superiority of any technique; subject to publication bias and selective outcome reporting
KLS cohorts and disease reviews	Define phenotype, episode characteristics, triggers, treatment, and interepisodic findings	Disease-informed perioperative considerations and baseline expectations	Do not evaluate perioperative interventions or outcomes
General perioperative guidelines and reviews	Support established medication, respiratory, anesthetic-depth, and neuromuscular-monitoring practice	General perioperative principles adapted to this population	Do not establish KLS-specific benefit
Mechanistic and analog-disorder literature	Provide a mechanistic account of anesthesia and emergence as modulation of arousal networks, and cautionary experience from related central hypersomnolence disorders	Biologic plausibility, hypothesis generation, and cautious extrapolation	Indirect; narcolepsy and KLS differ in phenotype and biology, and this literature should not be treated as clinical validation

Neurobiological rationale for anesthetic caution

Modern anesthetic neuroscience views general anesthesia as a reversible alteration of network connectivity, thalamocortical integration, cortical processing, and ascending arousal signaling rather than a uniform global suppression of brain activity [11-15]. Emergence from anesthesia involves coordinated recovery of multiple arousal systems, including orexinergic, dopaminergic, cholinergic, and noradrenergic pathways [11-17].

This framework is relevant to KLS because the disorder is considered a state-dependent dysfunction involving hypothalamic, thalamic, limbic, and frontotemporal networks rather than a fixed structural abnormality [1-8]. Neuroimaging studies have demonstrated abnormalities involving thalamic, hypothalamic, frontal, temporal, and associative networks during or around episodes, supporting a model of transient arousal-network dysregulation [5,6]. Although cerebrospinal fluid orexin levels are not consistently reduced in KLS and the disorder is distinct from narcolepsy, orexin pathways remain relevant because they contribute to arousal stability and to emergence from anesthesia [7,16].

These observations do not establish that anesthetic agents precipitate KLS recurrence. Instead, they provide a mechanistic rationale for reducing factors that may prolong central depression or obscure postoperative assessment. A conservative approach therefore includes avoiding unnecessary sedative exposure, using short-acting titratable agents when appropriate, minimizing long-acting opioids, preventing excessive anesthetic depth, and establishing a structured postoperative assessment plan before recovery begins.

Clinical considerations and proposed framework

Medication and Anesthetic Agent Considerations

Perioperative medication planning should include review of therapies used for hypersomnolence, recurrence prevention, psychiatric symptoms, pain, and other neurologic conditions. Lithium is used for episode prevention in selected patients with KLS and is included in current guidelines for central disorders of hypersomnolence, although the supporting evidence is observational [30-32]. Perioperative concerns include its narrow therapeutic range, renal clearance, electrolyte sensitivity, and potentiation of both depolarizing and nondepolarizing neuromuscular blockade. Reduced presynaptic acetylcholine synthesis or release and membrane-stabilizing effects have been proposed as mechanisms for this interaction [33,34]. Antiepileptic and psychotropic therapy may also be present for comorbid indications rather than for KLS itself, as in one published perioperative report [25], and should be reviewed on an agent-specific basis rather than treated as disease-specific KLS therapy.

Dexmedetomidine may be a useful selected adjunct because its alpha-2 adrenergic mechanism provides arousable sedation with relatively limited respiratory depression compared with many sedative-hypnotics [35-37]. In KLS, the rationale is pharmacologic and opioid/sedative sparing rather than evidence of recurrence prevention. Evidence for postoperative delirium reduction comes from a separate literature, chiefly trials in older non-KLS surgical populations [38,39], and should not be interpreted as demonstrating KLS-specific benefit.

Ketamine may provide opioid-sparing analgesia in selected patients. Consensus recommendations support its use as part of multimodal pain management, and experimental data suggest potential preservation of respiratory drive compared with deeper gamma-aminobutyric acid (GABA)ergic sedation [40,41]. However, ketamine is not without risk, and large procedural-sedation registry data associate it with a modest, dose-dependent increase in intraprocedural oxygen desaturation [42]. Psychotomimetic effects should also be considered because they may complicate postoperative assessment. Neither dexmedetomidine nor ketamine has been studied in KLS, and neither should be regarded as a preferred agent in this population.

Opioid minimization remains important because opioids can suppress ventilation, worsen sleep-disordered breathing physiology, and contribute to sedation that interferes with neurologic evaluation [18-20]. This does not preclude opioid use but supports consideration of regional techniques, multimodal analgesia, and short-acting opioids when necessary.

Processed electroencephalography may assist anesthetic titration and recognition of unnecessarily deep anesthesia in selected cases, as illustrated in some published KLS reports [22,23,25]. No KLS-specific index range, electroencephalographic signature, or outcome benefit has been established, and processed electroencephalography should be regarded as optional titration support rather than a therapeutic intervention [43].

When nondepolarizing neuromuscular blockers are used, quantitative monitoring and confirmation of adequate recovery before extubation are appropriate because residual weakness may confound neurologic and respiratory assessment. Current American Society of Anesthesiologists (ASA) guidance recommends quantitative monitoring and confirmation of a train-of-four ratio of at least 0.9 before extubation [44]. This is established general perioperative practice rather than a KLS-specific intervention, although it is particularly useful in this population, and lithium exposure may further prolong neuromuscular blockade [33,34]. Quantitative neuromuscular monitoring was not documented in every published KLS report in which a nondepolarizing agent was administered [25]. Table 3 summarizes chronic medication and anesthetic-agent considerations.

**Table 3:** Perioperative Medication and Anesthetic-Agent Considerations This table is an author-developed clinical aid and has not been prospectively validated. EEG: electroencephalography; KLS: Kleine-Levin syndrome; NSAID: non-steroidal anti-inflammatory drug; TIVA: total intravenous anesthesia; TOF: train-of-four

Category	Drug/Agent	Proposed approach	Rationale
Home medication	Stimulants / wake-promoting agents	Individualize with prescriber; document baseline alertness	May affect anesthetic needs; withholding may worsen hypersomnolence
Home medication	Lithium	Coordinate with prescriber; continue for minor cases, consider interruption for major surgery or fluid shifts	Narrow therapeutic index; may prolong neuromuscular blockade
Home medication	Antidepressants/antiepileptics/mood stabilizers	Usually continue; review agent-specific interactions	Abrupt cessation risks mood or seizure control
Home medication	Chronic opioids/gabapentinoids	Avoid withdrawal; minimize added sedative stacking	Complicates distinguishing sedation from recurrence
Anesthetic technique	Regional/neuraxial/local	Consider as primary strategy when clinically appropriate	Reduces systemic hypnotic and opioid burden
Anesthetic adjunct	Dexmedetomidine	Selected adjunct for arousable sedation, opioid sparing	Limited respiratory depression; no direct KLS data
Anesthetic adjunct	Low-dose ketamine	Opioid-sparing adjunct in selected patients	May preserve ventilatory drive; risk of desaturation/dysphoria
Anesthetic agent	Propofol	Induction, or short titratable TIVA	Rapid, controllable; used successfully in published KLS cases
Anesthetic agent	Remifentanil/short-acting opioid	Short intraoperative bridge when needed	Minimal accumulation vs. long-acting opioids
Anesthetic agent	Volatile anesthetics	Maintenance at appropriate depth when TIVA not used	Uneventful in two reports; avoid excessive depth
Anesthetic agent	Neuromuscular blocking agents	Use as indicated; quantitative monitoring, TOF ≥ 0.9 before extubation	Residual weakness can mimic impaired emergence
Monitoring	Processed EEG	Optional aid to anesthetic titration	No KLS-specific benchmark established
Anesthetic: minimize	Benzodiazepines/long-acting sedatives	Minimize routine premedication; consider non-pharmacologic anxiolysis	Prolongs recovery; impairs postoperative assessment
Anesthetic: minimize	Long-acting opioids/sedating polypharmacy	Prefer regional/local techniques, acetaminophen/NSAIDs, ketamine, short-acting opioids	Can mimic or mask KLS hypersomnolence

Proposed Perioperative Management Framework

**Figure 2:**
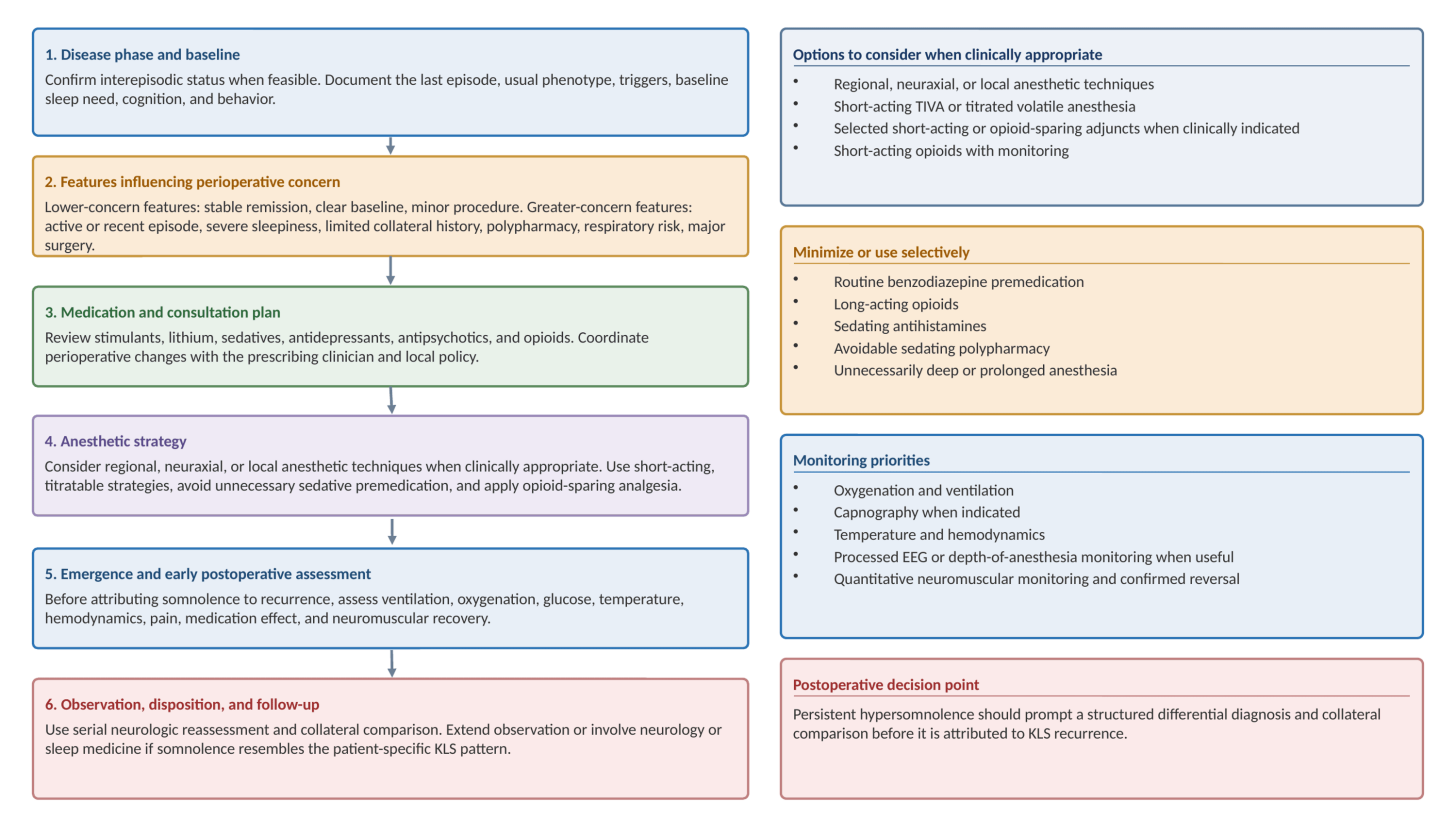
Proposed Perioperative Management Framework for Kleine-Levin Syndrome This conditional, mechanism-informed framework organizes disease-phase and baseline assessment, features influencing perioperative concern, medication review and consultation, anesthetic planning, physiologic monitoring, emergence assessment, postoperative observation, disposition, and follow-up. Persistent postoperative hypersomnolence should prompt a structured differential diagnosis and comparison with the patient’s usual KLS phenotype before recurrence is presumed. The framework has not been prospectively validated and should not be interpreted as a formal clinical guideline. EEG: electroencephalography; KLS: Kleine-Levin syndrome; TIVA: total intravenous anesthesia

The framework starts with disease-phase classification (Figure 2). When clinically feasible, elective surgery may be scheduled during a stable interepisodic period to facilitate baseline assessment and interpretation of postoperative recovery [21-25]. When a patient is in an active episode, deferral may be considered after individualized assessment of procedural urgency, current disease phenotype, and available postoperative monitoring; no data demonstrate that postponement alters recurrence risk. For urgent or emergent surgery, the emphasis shifts to documentation of baseline status, collateral history, minimization of avoidable sedatives, and planning for extended recovery monitoring. Table 4 summarizes clinical features that may influence perioperative concern; these features are not a validated predictive score.

**Table 4:** Clinical Features and Preoperative Risk Stratification in KLS These features are unvalidated clinical considerations intended to support individualized perioperative planning. They do not constitute a predictive score or estimate the probability of perioperative complications or KLS recurrence. CNS: central nervous system; ICU: intensive care unit; KLS: Kleine-Levin syndrome; OSA: obstructive sleep apnea; KLS: Kleine-Levin syndrome

Assessment domain	Perioperative relevance	Lower concern features	Greater concern features
Disease phase	Active-episode symptoms can mimic delayed emergence or delirium	Clearly interepisodic; baseline documented	Active episode; no assessable baseline
Cognitive/behavioral baseline	KLS symptoms overlap with emergence delirium	Documented baseline with collateral history	Unclear baseline; no collateral available
Chronic CNS-active medications	May mimic recurrence or alter anesthetic requirements	No lithium or sedating polypharmacy	Lithium with renal/fluid concern; chronic sedatives; high opioid tolerance
Airway/sleep-disordered breathing	Opioids/sedatives prolong somnolence, depress ventilation	No known OSA; reassuring airway exam	Untreated OSA, obesity, difficult airway, hypercapnia risk
Autonomic/thermoregulatory features	Dysautonomia has been reported during episodes	No autonomic symptoms reported	Reported dysautonomia or thermoregulatory instability
Procedure and anesthetic exposure	Longer exposure increases postoperative diagnostic ambiguity	Minor procedure; regional technique feasible	Long surgery, neurosurgery, or ICU-level recovery
Postoperative observation capacity	Serial reassessment needed to separate drug effect from symptoms	Recovery unit able to reassess serially	Limited monitoring capacity

The preoperative assessment should include the date and duration of the last episode, the usual episode phenotype, observed triggers, baseline sleep requirement and cognition, current stimulant or lithium use, other psychoactive medications, substance use, and coexisting sleep-disordered breathing. Input from the family or caregiver is especially valuable because patients may recall prior episodes incompletely [1-4,9]. Routine benzodiazepine premedication is best minimized unless specifically indicated. In one published KLS case, parental presence and familiar music were used as non-pharmacologic anxiolytic measures, with no separate sedative premedication described [25].

Intraoperatively, the plan should focus on titratability and postoperative interpretability. Regional, neuraxial, or local anesthetic techniques may be considered when clinically appropriate, accepted, and not contraindicated [21]. When general anesthesia is required, a short-acting, titratable intravenous or volatile technique may be selected according to the procedure, patient comorbidities, and clinician judgment; both approaches have been used successfully in individual cases [22,23,25]. Unnecessarily deep anesthesia should be avoided, and normoxia, normocapnia, normothermia, and hemodynamic stability maintained [11-15]. Analgesia should be multimodal and opioid-sparing, using regional or local techniques, acetaminophen and nonsteroidal anti-inflammatory drugs (NSAIDs) when safe, and low-dose ketamine in selected patients, with long-acting opioids minimized [18-20,40,41]. Processed electroencephalography should be read as a trend and depth-avoidance tool rather than a KLS-specific endpoint; when neuromuscular blocking agents are used, quantitative monitoring and confirmed recovery are appropriate, since residual paralysis can mimic impaired emergence, especially after lithium exposure [33,34,43,44].

Emergence should be treated as an organized neurologic handover rather than a binary extubation event. Parameters documented by the team before the patient leaves the operating room should include eye opening, command following, ventilatory adequacy, neuromuscular recovery, and the degree of interaction relative to baseline, together with explicit communication of the expected emergence trajectory, the drugs and reversal administered, the documented baseline cognition, and the typical episode phenotype for that patient. Family-confirmed return toward baseline, when available, is helpful but should not delay management of urgent airway, respiratory, metabolic, or neurologic causes of depressed sensorium.

Postoperative Diagnostic Framework

Postoperative care should focus on serial reassessment rather than on a single diagnosis such as delayed emergence or recurrence. On arrival in the recovery unit, the team should check airway patency, ventilation, oxygenation, temperature, glucose when indicated, hemodynamics, pain, medication timing, neuromuscular recovery, and level of interaction, with reassessment on arrival and serially at clinically appropriate intervals according to the patient’s trajectory and local recovery-unit policy, in order to separate improving pharmacologic sedation from persistent or evolving disease-like symptoms.

Terminology should distinguish four situations that are frequently conflated: postoperative somnolence, which is common and usually pharmacologic; a KLS-like postoperative phenotype, in which the pattern resembles the patient’s usual episodes; a suspected KLS recurrence, in which that pattern persists without an alternative explanation; and a confirmed KLS episode. During the immediate postoperative period, persistent somnolence or neurobehavioral change should be described as a KLS-like phenotype or suspected recurrence rather than a confirmed KLS episode. Confirmation requires persistence consistent with the recognized episode duration and phenotype, together with exclusion of pharmacologic, respiratory, metabolic, neurologic, psychiatric, and other medical explanations. Because current diagnostic criteria define episodes as persisting from two days to several weeks [27], a few hours of recovery-unit somnolence cannot satisfy the definition.

A suspected KLS recurrence should accordingly be considered only as a diagnosis of exclusion, after reversible causes of decreased responsiveness have been evaluated. These include residual hypnotic, opioid, or neuromuscular effect, hypercapnia, hypoxemia, hypoglycemia, hypothermia, electrolyte disturbance, medication interaction, pain, infection, urinary retention, seizure, stroke, and delirium. When somnolence persists beyond the expected pharmacologic window, approximates the patient’s prior episodes, and occurs with stable ventilation and no alternative explanation, neurology or sleep-medicine consultation is reasonable. Patients and families should receive discharge counseling on recurrence warning signs, sleep protection, medication resumption, and when to seek urgent care. Table 5 presents a structured interpretation of common postoperative presentations.

**Table 5:** Postoperative Neurobehavioral Interpretation Framework This table is an author-proposed clinical aid rather than a validated protocol. KLS: Kleine-Levin syndrome

Presentation	Most important differential interpretation	Suggested evaluation and response
Prolonged somnolence with gradual improvement	Residual anesthetic, opioid, benzodiazepine, or dexmedetomidine effect; sleep debt; pain-related fatigue	Review the anesthetic record, drug timing, renal and hepatic factors, ventilation, temperature, glucose, and the pain plan; avoid premature attribution to recurrence
Somnolence with hypoventilation, desaturation, or hypercapnia	Opioid effect; residual neuromuscular blockade; obstructive sleep apnea; airway obstruction	Treat airway and ventilation first; consider opioid reversal when appropriate; verify quantitative train-of-four recovery and reversal status
Weakness, poor grip strength, or inadequate ventilation without marked sedation	Residual neuromuscular blockade, potentially prolonged by lithium	Perform quantitative train-of-four assessment and confirm a ratio of at least 0.9; administer further reversal as indicated before attributing findings to the disease
Somnolence with abnormal laboratory values or abnormal temperature	Hypoglycemia, electrolyte disturbance, hypothermia, or another metabolic cause; dysautonomic thermoregulatory instability	Check glucose, electrolytes, and temperature; correct abnormalities and reassess before considering the disease
Confusion, agitation, derealization, or unusual behavior	Emergence delirium; pain; hypoxia; anticholinergic or ketamine effect; the patient’s baseline KLS symptom pattern	Use calm reorientation, treat physiologic causes, obtain collateral history, and avoid reflexive benzodiazepine escalation
Somnolence or agitation with fever, tachycardia, discomfort, or bladder distension	Infection, untreated pain, or urinary retention	Examine for a source; assess the bladder; treat pain and infection, then reassess the neurologic picture
Focal deficit, seizure activity, or abrupt unexplained deterioration	Neurologic complication such as stroke or seizure	Escalate urgently; obtain neurologic assessment and imaging as indicated; do not attribute to KLS
Stable vital signs with persistent hypersomnolence resembling prior episodes	Suspected KLS recurrence or KLS-like postoperative phenotype, once pharmacologic and other causes are less likely	Extend observation; involve neurology or sleep medicine; protect the sleep-wake schedule; document phenotype and duration before describing the event as a confirmed episode
Normal recovery trajectory	Expected emergence and postoperative fatigue	Resume the individualized chronic medication plan; advise the patient to report delayed recurrence over subsequent days

Limitations and future directions

The principal limitation is the absence of prospective or comparative perioperative data in KLS. The direct evidence comprises five case-level publications involving heterogeneous procedures, anesthetic techniques, disease phases, reporting depth, and follow-up durations [21-25]. Although all five described favorable immediate perioperative courses, these observations cannot establish complication or recurrence rates, comparative safety, or superiority of any anesthetic technique. The evidence is susceptible to publication bias, selective outcome reporting, variable diagnostic-era criteria, and incomplete or non-standardized follow-up. Descriptive JBI appraisal [26] indicated generally adequate case reporting but does not overcome the inherent limitations of uncontrolled single-patient observations.

A second limitation is the extent of indirect inference. Many proposed considerations are adapted from general anesthetic pharmacology, sleep-wake neurobiology, pain management guidance, psychotropic medication literature, neuromuscular monitoring guidelines, and perioperative studies of other central hypersomnolence disorders [11-20,32-47]. These sources provide biologic or clinical rationale but do not demonstrate KLS-specific benefit. Narcolepsy should not be treated as interchangeable with KLS because the disorders differ in phenotype and disease biology [45,46]. The available survey data in central disorders of hypersomnolence included very limited KLS representation and are treated here as analog rather than disease-specific evidence [47].

Finally, the evidence map, the features influencing perioperative concern, the medication considerations, and the postoperative evaluation framework were developed by the authors and have not been prospectively validated. Terms such as "consider," "minimize," and "when clinically appropriate" indicate conditional clinical reasoning rather than formal recommendations, and the figures should be read in the same way. Procedure urgency, patient preference, comorbidities, local policy, and multidisciplinary consultation remain central to individual decisions.

The next step is to better describe perioperative outcomes rather than to recommend specific approaches. A multicenter registry could capture disease phase, timing of the last episode, chronic medication use, anesthetic technique, depth monitoring, neuromuscular blocker exposure, opioid dose, emergence trajectory, recovery unit duration, postoperative sleep pattern, and 30-day recurrence, allowing the field to move beyond anecdote. Prospective observational studies could evaluate opioid-sparing pathways, although randomized trials may be difficult given the rarity of the condition. Standardized reporting of the variables listed in Table 2 would make future case-level publications more comparable. Detailed case reports and mechanistic studies incorporating electroencephalography, autonomic measures, inflammatory markers, and orexin-related measures may further clarify disease recurrence.

## Conclusions

Perioperative management of KLS is best conceptualized as a problem of arousal network vulnerability, for which postoperative diagnostic clarity can be prioritized, rather than as a contraindication to general anesthesia. Five patient-level reports, comprising one neuraxial and four general anesthesia cases, indicate that both approaches have been delivered successfully in individual patients. This evidence establishes feasibility; however, it is too limited to define a guideline or a preferred technique for all cases.

A defensible interim strategy is to consider scheduling elective procedures during stable interepisodic periods when clinically feasible, define baseline cognition before anesthetic exposure, minimize unnecessary sedative premedication, consider regional, neuraxial, or local anesthetic techniques and opioid-sparing multimodal analgesia when clinically appropriate, use short-acting titratable agents when general anesthesia is required, and apply anesthetic depth and quantitative neuromuscular monitoring where indicated. Persistent postoperative somnolence should not be labeled KLS recurrence until residual anesthetic or opioid effect, hypoventilation or hypercapnia, metabolic or temperature disturbance, residual neuromuscular blockade, delirium, infection, pain, urinary retention, and neurologic causes have been considered. This framework should be regarded as conditional and hypothesis-generating until multicenter perioperative data become available.

## Appendices

Descriptive JBI appraisal of the five direct perioperative management reports

The JBI Critical Appraisal Checklist for Case Reports was applied descriptively to characterize reporting completeness [26]. “Yes” indicates that the domain was adequately reported; “Unclear” indicates insufficient detail to judge. No numerical summary score or exclusion threshold was applied, and all five reports were retained [21-25].

**Table 6:** : Descriptive JBI Appraisal of the Five Direct Perioperative-Management Reports

Appraisal domain	Rehman et al. [21]	Ben-Menachem and Winder [22]	Fujita and Mizuta [23]	Rajaleelan et al. [24]	Chowdhury et al. [25]
Demographics	Yes	Yes	Yes	Yes	Yes
History/timeline	Yes	Yes	Yes	Yes	Yes
Clinical condition	Yes	Yes	Yes	Yes	Yes
Diagnostic assessment	Unclear	Yes	Yes	Yes	Yes
Intervention/treatment	Yes	Yes	Yes	Yes	Yes
Post-intervention status	Yes	Yes	Yes	Yes	Yes
Adverse/unanticipated events	Yes	Yes	Yes	Yes	Yes
Takeaway lessons	Yes	Yes	Yes	Yes	Yes
Overall appraisal	Include	Include	Include	Include	Include

The appraisal identified incomplete reporting of disease-specific diagnostic assessment in the earliest report [21] but otherwise generally complete description of patient characteristics, anesthetic interventions, postoperative outcomes, and clinical lessons. The checklist was used to describe limitations in reporting, not to infer comparative certainty of evidence.
